# Combinatorial Enhancement of Aging Resistance in High-Content Crumb Rubber Asphalt via Warm-Mix Additives: Rheological and Microstructural Insights

**DOI:** 10.3390/ma18225161

**Published:** 2025-11-13

**Authors:** Jia Guo, Xiang Han, Yuhan Shi, Yue Xiao, Lan Wang, Zhendong Liu

**Affiliations:** 1 School of Materials Science and Engineering, Chang’an University, Xi’an 710064, China; guojia@chd.edu.cn (J.G.); 2023903805@chd.edu.cn (X.H.); 13302948896@163.com (Y.S.); 17349300334@163.com (Z.L.); 2 School of Civil Engineering, Inner Mongolia University of Technology, Hohhot 010051, China

**Keywords:** high-content crumb-rubber-modified asphalt, warm-mix additive, aging resistance, rheological deterioration, micromechanical stabilization

## Abstract

Conventional rubber-modified asphalt typically suffers from low rubber content and requires high construction temperatures. This study developed a warm-mix high-content crumb-rubber-modified asphalt (CRMA) with an increased rubber particle content of over 20%; moreover, the optimization of the warm-mixing agent was determined. Its rutting and cracking resistance performances were investigated using a dynamic shear rheometer (DSR) and a bending beam rheometer (BBR). Fourier Transform Infrared (FTIR) and Atomic Force Microscopy (AFM) were used to characterize the aging resistance and microstructural characteristics. The key findings revealed that the optimal dosage of the SDYK-type warm-mix additive (SDYK; a surfactant used to improve the high-temperature stability, low-temperature crack resistance, and anti-aging performance of asphalt) was 0.6% for high-rubber-content CRMA. The combination of warm-mix additives and rubber granules enhanced the aging resistance and elasticity of the asphalt while also contributing to an increase in chemical functional group indicators. The decrease in both the aliphatic chain index and branched alkane index of CRMA indicates that the warm-mix agent and the rubber additive enhanced the aging resistance of the asphalt. The warm-mix agent reduced the roughness of the asphalt, counteracting the roughness-enhancing effect of crumb rubber. This was attributed to the lubrication effect induced by the water film during the mixing process, which promotes a more uniform distribution of the rubber crumb network. This research established a theoretical and experimental basis for the application of high-rubber-content CRMA in large-temperature-difference regions.

## 1. Introduction

In recent years, the increase in the number of motor vehicles in China has led to a significant rise in waste tires [1,2]. The recycling rate of waste tires is less than 60%, contributing to the increasing severity of environmental pollution [3,4,5,6]. In addressing this issue, pavement engineers have demonstrated the technical feasibility of using CRMA as a sustainable alternative to asphalt. This material serves as an eco-friendly construction solution [7,8], combining waste tire reuse with enhanced pavement performance [9].

CRMA has been investigated extensively by numerous scholars for its multifunctional advantages [10,11]. Ghazi G. Al-Khateeb et al. [12] reported that crumb rubber could improve the anti-rutting and anti-fatigue properties of the asphalt binder. Zhou Tao et al. [13] studied the physical and chemical changes in modified asphalt under aging and prepared modified asphalt using microwave-activated crumb rubber. It was found that the microwave process made the swelling reaction of the crumb rubber more intense. I.M. Ibrahim [14] researched rubberized asphalt made from waste crumb rubber pre-treated with gamma rays. The study showed that rubberized asphalt made using pre-treated powder had better high- and low-temperature performance and aging resistance than ordinary rubberized asphalt. It was found that asphalt aging produced many polar groups, like carbonyl and sulfoxide groups [15]. This led to increased molecular aggregation in asphalt. It also increased the viscosity and modulus of the asphalt but decreased its ductility.

Despite these benefits, traditional CRMA faces two critical limitations: (1) low rubber content (typically 15–25% by binder weight [10,16,17]) restricts the rates of waste tire utilization; (2) the high construction temperatures (180–200 °C) necessitated by rubber devulcanization leads to excessive energy consumption and emissions, limiting its widespread use [18].

High-rubber-content-modified asphalt is produced by mixing asphalt, substantial amounts of waste tire crumb rubber, and various additives at high temperatures under shear conditions. “High rubber content” typically refers to CRMA mixtures containing 30% to 50% rubber particles by weight. According to industry standards, conventional rubber-modified asphalt generally contains 15% to 25% rubber content [19,20,21]. This approach not only increases the recycling rate of waste tire rubber but also enhances asphalt performance, such as enhanced rutting resistance and increased flexibility at low temperatures [11,22]. Therefore, high-rubber-content-modified asphalt is a research hotspot in the pavement industry [11,23,24].

Rubber-modified asphalt requires elevated mixing temperatures between 180 °C and 200 °C during construction. This high-temperature process is necessary for rubber devulcanization but leads to increased energy consumption and emissions [25,26]. The application of warm-mixing technology reduces the construction temperature of CRMA mixtures. It decreases rutting resistance during pavement service, thereby extending its service life [27,28]. In environments with large annual temperature differences, the performance of road asphalt materials can rapidly deteriorate. During summer, high temperatures reduce the stiffness modulus, causing insufficient rutting resistance and leading to deformation. In winter, low temperatures embrittle the material, decreasing crack resistance and resulting in thermal cracking [29]. The addition of warm-mix agent improves the high-temperature rutting resistance, rheological properties, and low-temperature cracking resistance of asphalt [30].

In the early stages, most researchers employed DSR and BBR tests to investigate the high- and low-temperature rheological properties of asphalt. For example, Wang [31] evaluated the rheological behavior of asphalt using DSR and BBR tests and found that rubber-modified asphalt exhibited excellent performance under high- and low-temperature conditions. Compared to neat asphalt, CRMA also demonstrated significantly improved fatigue resistance. However, it is difficult to accurately characterize the high- and low-temperature rheological properties of asphalt using only macroscopic tests. In recent years, FTIR and AFM were employed to analyze the chemical structures of asphalt, which exhibited correlations with its rheological properties. By combining microscopic and macroscopic tests, a more accurate analyses of the high- and low-temperature rheological behavior of asphalt can be achieved. Wang [32] investigated the corrosion resistance of CRMA at low temperatures and its correlation with chemical properties through BBR and FTIR tests. The results showed that the functional groups of asphaltenes and aromatics were significantly correlated with the low-temperature performance of the CRMA binder. Zhang [33] conducted an analysis of the rheological and micromechanical properties of warm-mix CRMA using AFM and infrared spectroscopy, in combination with DSR and BBR tests.

To address the low crumb rubber content in traditional CRMA, this study added higher doses (above 15–20%) of rubber crumb to overcome the limitations. Furthermore, an SDYK warm-mix additive was incorporated to enhance CRMA’s applicability in regions with significant temperature variations. By using short- and long-term aging conditions, this research investigated the high- and low-temperature rheological properties of warm-mix high-rubber-content-modified asphalt through a combination of macroscopic and microscopic testing methods.

## 2. Materials and Research Methodologies

### 2.1. Raw Materials

Pen 90 base asphalt was selected for this study (manufactured Panjin Zhongrui Asphalt Co., Ltd. in Panjin City, China). The performance indices of 60 mesh rubber particles are shown in Table 1. The warm-mix agent is an SDYK surfactant developed by Shandong Academy of Transportation Sciences, in Shandong Province, China. It is a dark-yellow viscous liquid at specific concentrations. It can be used to directly improve the high-temperature stability, low-temperature crack resistance, and anti-aging performance of asphalt. This additive is suitable for various types of asphalt mixtures [28].

### 2.2. Experimental Design

**Figure 1:**
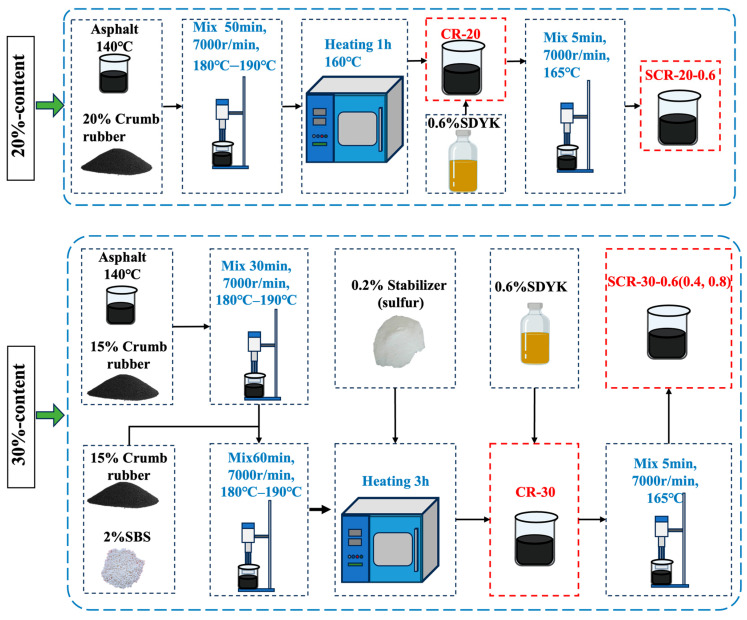
The preparation method for the asphalt in this study.

The modifier was 60 mesh crumb rubber particles, with the crumb rubber content accounting for 20% and 30% of the asphalt mass. The dosages of warm-mix agent were 0.4%, 0.6%, and 0.8% of the mass of the CRMA, respectively. The preparation method of the 20% CRMA was previously studied by our research group; the findings suggested adding 0.6% of warm-mix additives to the asphalt with 20% rubber content [34]. The preparation process of the 30% content warm-mix CRMA (SCR-30-0.6) and the 20% content warm-mix CRMA (SCR-20-0.6) is shown in Figure 1. The representative symbols of the prepared asphalt are shown in Table 2.

The preparation process of SCR-20-0.6 was as follows: (1) The base asphalt was heated to 140 °C, followed by adding 20% crumb rubber. The temperature was then raised to 180–190 °C with continuous stirring for 50 min. (2) Then, the mixture was cured in a 160 °C oven for 1 h to obtain the CR-20 matrix asphalt. (3) The CR-20 matrix was stirred at 165 °C while adding 0.6% SDYK warm-mixing agent. Mixing continued for 5 min to produce the final product.

The preparation of SCR-30-0.6 followed similar steps as those used for SCR-20-0.6, with the following modifications: (1) Staged material addition: 15% activated crumb rubber was first mixed for 30 min, followed by adding another 15% crumb rubber and 2% SBS (Styrene–Butadiene–Styrene triblock copolymer, a thermoplastic elastomer with a typical molecular weight ranging from 100,000 to 300,000 g/mol and a styrene content of approximately 30 wt%) for an additional 30 min. SBS acted as a stabilizer in asphalt binders. (2) Shearing parameters: The shearing temperature was increased to 180–190 °C, and 0.2% SBS stabilizer was added after shearing. (3) Extended curing: The curing time was prolonged to 3 h. The warm-mixing agent-incorporation process remained identical to that used for SCR-20-0.6.

Key improvements in SCR-30-0.6 included the staged addition of crumb rubber, enhanced shearing temperature, and extended curing to optimize compatibility and stability. The preparation methods used for SCR-30-0.4 and SCR-30-0.8 were identical to those used for SCR-30-0.6. The only difference lies in the dosage of SDYK used, which was 0.4% for SCR-30-0.4 and 0.8% for SCR-30-0.8.

### 2.3. Research Methodologies

This study systematically elucidates the multi-scale performance evolution mechanism of warm-mix high-rubber-content CRMA. Firstly, DSR and BBR tests were employed to quantify the trends in the high-temperature deformation resistance and low-temperature cracking resistance of the asphalt; this was performed in order to determine whether the combined warm-mix and high-content modification led to improvements in the macroscopic mechanical performance. FTIR was utilized to focus on the variation in chemical functional group indices, allowing us to verify the evolution of the molecular structure during aging and to support the chemical reaction mechanisms underlying the macroscopic performance changes. AFM was applied to reveal nanoscale variations in surface morphology, roughness, adhesion force, and DMT modulus, aiming to uncover the microscopic mechanisms by which the warm-mix additive improves structural uniformity and compatibility. The comparative tests before and after aging demonstrated the combinatorial mechanism of the warm-mix technology and high-content crumb rubber in delaying aging and stabilizing the asphalt structure. Based on these tests, this study recommends an optimal warm-mix additive dosage of 0.6%, and systematically reveals the combinatorial evolution mechanism of warm-mix high-rubber-content CRMA in terms of aging resistance, high- and low-temperature performance, and microstructural stability. In the research, the number of samples is greater than or equal to 3.

**Figure 2:**
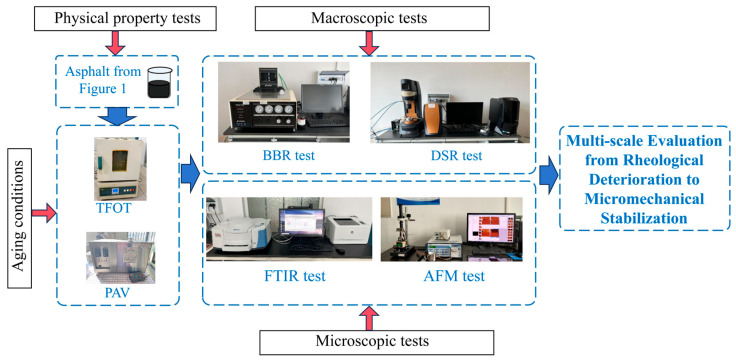
Research flowchart.

The research flowchart was illustrated in Figure 2.

#### 2.3.1. Physical Property of Asphalt

The penetration, softening point, and ductility of the asphalt were evaluated following ASTM D5 [35], D36 [36], and D113 [37], respectively. The rotational viscosity was evaluated following ASTM D4402 [38], with a temperature of 175 °C, using Spindle 27 (Brookfield Engineering Labs, Inc., Middleboro, MA, USA) at a speed of 20 rpm.

#### 2.3.2. Accelerated Aging in Laboratory

The short-term aging was simulated using a SYD-0610 Asphalt rotary film oven (Shanghai Changji Geological Instrument Co., Ltd., Shanghai, China). The simulation followed ASTM D1754 standard [39]. The sample was heated at 163 ± 1 °C for 5 h.

The long-term aging samples were simulated with a Prentex Model 9500 pressure aging vessel (PAV, Prentex Alloy Fabricators, Dallas, TX, USA). The simulation followed ASTM D6521 standard [40]. The pressure was maintained at 2.1 MPa ± 0.1 MPa, the temperature was set to 100 °C ± 0.5 °C, and the aging duration was 20 h.

#### 2.3.3. Dynamic Shear Rheological Test

The DSR test was evaluated following ASTM D7552-09 [41]. The Discovery HR-2 dynamic shear rheometer (TA Instruments, New Castle, DE, USA) was employed in this study. The diameter of the parallel plates was 25 mm ± 0.05 mm, and the gap size was 1 mm. Temperature sweeps were used to study the high-temperature rheology of high-rubber-content CRMA. The temperature sweeps used temperatures ranging from 28 °C to 82 °C, with intervals of 6 °C. All tests were conducted under a constant preload pressure of 120 kPa and a fixed angular frequency of 10 rad/s. The strain amplitude was maintained at 1%.

#### 2.3.4. Bending Beam Rheological Test

The BBR test was evaluated following ASTM D6648 [42]. BBR was employed to measure the creep properties of asphalt at low temperature. A Cannon Instruments (USA) BBR system was utilized in this study. The experimental standard incorporated three test temperatures (−18 °C, −24 °C, and −30 °C). All mechanical loading sequences maintained a standardized load application duration of 60 s.

#### 2.3.5. Fourier Transform Infrared Spectroscopy Test

The infrared spectrum test was to analyze the functional groups of asphalt. Nicolet iS10 (Thermo Fisher Scientific, Waltham, MA, USA) was used to test CRMA. The spectral acquisition parameters were set to 32 cumulative scans with 4 cm^−1^ resolution, spanning a wavenumber range of 400–4000 cm^−1^. This test was evaluated following ASTM E1252 [43].

#### 2.3.6. Atomic Force Microscope Test

AFM was employed to study the surface morphology and mechanical properties of asphalt. In this experiment, the tapping mode suitable for soft viscous materials was adopted, the scanning range was 20 μm × 20 μm, the scanning frequency was 0.977 HZ, and the temperature was 25 °C. This test was evaluated following ASTM E2859-11 [44].

## 3. Results and Discussion

### 3.1. Optimization of Warm-Mixing Agent

The dosage of warm-mix additives for high-rubber-content CRMA was determined based on materials technical specifications and its high-/low-temperature performance.

The results for the three key performance indicators (penetration, softening point, and ductility) and viscosity for the asphalt types are summarized in Table 3. SCR-30-0.6 exhibited the lowest penetration value, the highest softening point, the highest ductility, and the minimum viscosity among the high-rubber-content asphalt blends. This suggested that the SCR-30-0.6 composite exhibits superior high-temperature stability, excellent low-temperature crack resistance, and enhanced rutting resistance.

**Figure 3:**
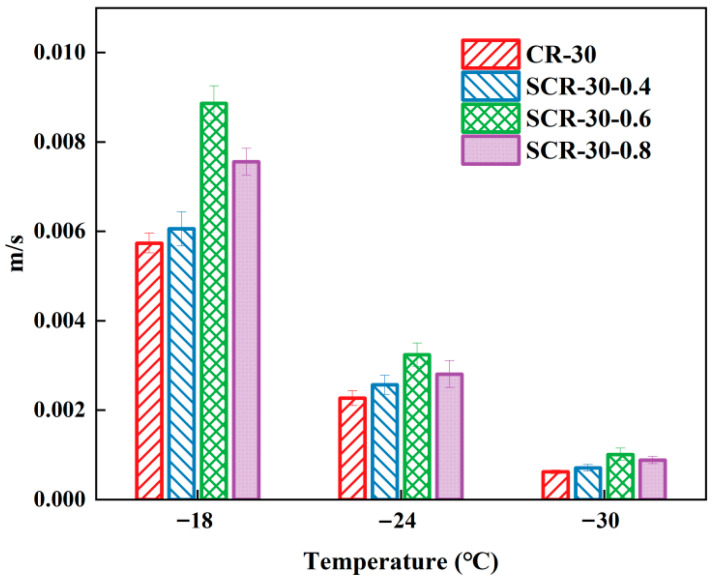
The m/S of high-rubber-content CRMA.

**Figure 4:**
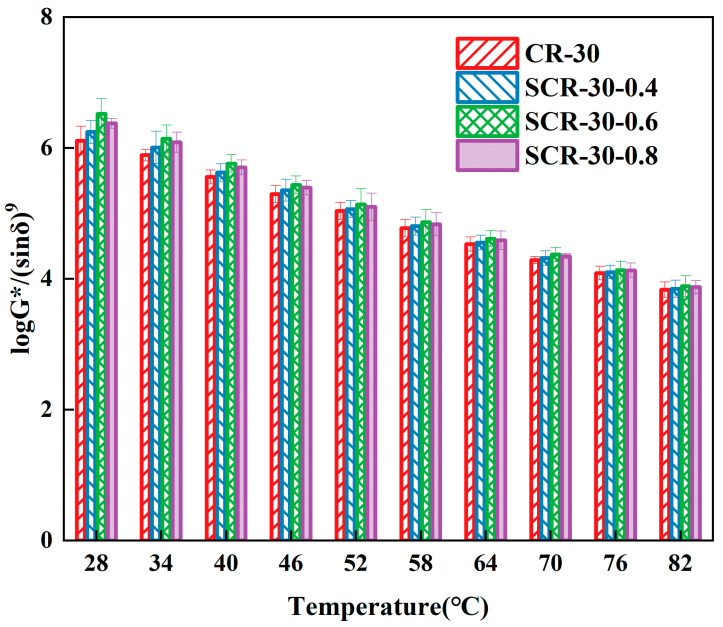
The improved rutting factor of high-rubber-content CRMA.

The bending beam rheometer (BBR) test evaluates material crack resistance through bending beam deformation characteristics. It measures beam deformation and stress to analyze asphalt’s rheological properties. The m/S index derived from BBR tests is used to assess asphalt’s low temperature performance, with a higher value indicating better performance [45,46]. This method determined the optimal warm-mix additive dosage for high-content rubberized asphalt in low-temperature conditions. The improved rutting factor G*/sin δ based on temperature sweeps can better evaluate the high-temperature deformation resistance of warm-mix CRMA [47]. Therefore, this study employed this formula to investigate the high-temperature deformation resistance of asphalt. The larger the improved rutting factor G*/sin δ, the better the high-temperature deformation resistance. The m/S of high-rubber-content CRMA was presented in Figure 3, and the improved rutting factor was demonstrated in Figure 4.

From Figure 3, we can see that the m/S of all four asphalt types decreased with temperature reduction, indicating reduced low-temperature cracking resistance. Under the test temperature, the m/S values of SCR-30-0.6 were the largest. This showed that SCR-30-0.6 had the best crack resistance at low temperature, so it is recommended that the optimal SDYK mixture content of high-rubber-content CRMA at low temperature is 0.6%. The m/S values of SCR-30-0.4, SCR-30-0.6, and SCR-30-0.8 were all higher than those of CR-30. This demonstrates that adding the SDYK warm-mix agent enhances the low-temperature cracking resistance of CRMA. This improvement was attributed to SDYK’s cationic surfactant properties, and after interacting with the active site (-CH_2_) n branch chain of asphaltene, the small molecules of flexible branch chain increased, enhancing the low-temperature cracking resistance [48,49].

From Figure 4, the improved rutting resistance factors of all four asphalt types progressively decreased with increasing temperature. The improved rutting factors of SCR-30-0.4, SCR-30-0.6, and SCR-30-0.8 were all greater than those of CR-30, indicating that the addition of SDYK improved the high-temperature deformation resistance of CRMA. This is due to the addition of SDYK made the rubber better dissolve in the asphalt, and the void of the asphalt is filled, resulting in the increase in internal friction during molecular movement, thus reducing the phase Angle of the asphalt and increasing the complex modulus [50]. The improved rutting factor of SCR-30-0.6 was the highest at all test temperatures, so it was recommended that the optimal SDYK mixture content of high-rubber-content CRMA at high temperature is 0.6%.

Therefore, based on the comprehensive m/S and improved rutting factor indicators, this article recommends a warm-mix agent dosage of 0.6% for high-rubber-content CRMA. So that SCR-30-0.6 was selected as the warm-mixed high-rubber-content CRMA for the following study.

### 3.2. Improvement of Cracking Resistance

**Figure 5:**
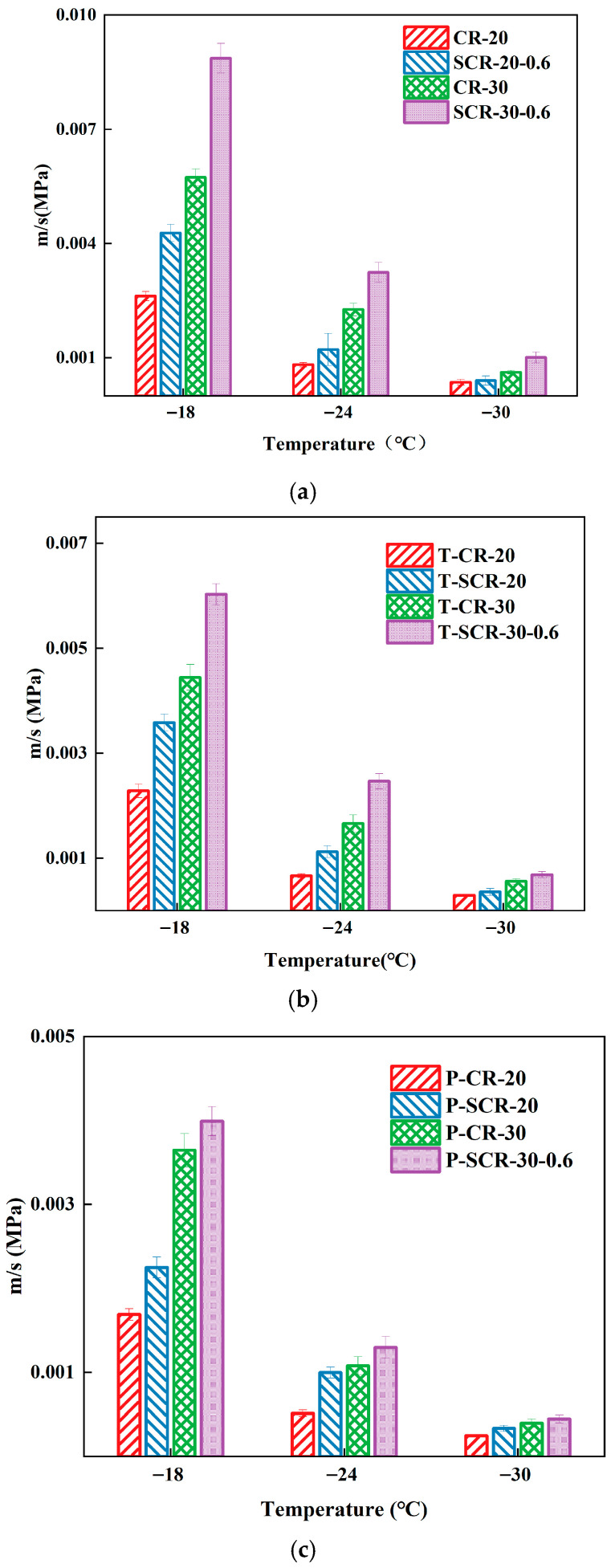
The m/S of high-rubber-content CRMA before and after aging. (**a**) Pre-aging; (**b**) short-term aging; (**c**) long-term aging.

The low-temperature cracking resistance of asphalt was evaluated utilizing the m/S parameter outlined in Section 3.1. The m/S values of high-rubber-content CRMA across aging conditions are shown in Figure 5. The exhibited trends confirmed the rheological degradation of the materials with aging.

From Figure 5a–c, as indicated by the vertical coordinates, we can see that the m/S values of asphalt exhibited a consistent decreasing trend with progressive aging levels. This indicated that, as the aging process intensifies, the low-temperature crack resistance of asphalt decreases. This trend mechanistically corresponds to the progressive embrittlement of the asphalt matrix, ultimately manifesting as compromised stress relaxation capabilities and diminished resistance to thermally induced fracture propagation at subzero temperatures.

Figure 5a shows the m/S values of CR-20, SCR-20-0.6, CR-30, and SCR-30-0.6 under pre-aging. The m/S value of SCR-30-0.6 was the highest, followed by CR-30. The addition of more crumb rubber and warm-mix agent improved the low-temperature crack resistance of asphalt. From Figure 5b, at the test temperature, the m/S size relationship of the four kinds of asphalt after short-term aging was T-SCR-30-0.6 > T-CR-30 > T-SCR-20-0.6 > T-CR-20. The low temperature performance of T-SCR-30-0.6 after short-term aging was the best, followed by T-CR-30. The addition of more rubber and SDYK enhances the low-temperature crack resistance of asphalt during short-term aging. As shown in Figure 5c, the m/S size order of asphalt after long-term aging was P-SCR-30-0.6 > P-CR-30 > P-SCR-20-0.6 > P-CR-20, where P-SCR-30-0.6 displayed the best low-temperature performance, followed by P-CR-30. The incorporation of more rubber and SDYK improves low-temperature crack resistance during long-term aging as well. This is because, as the asphalt undergoes aging, the crumb rubber particles generate numerous cracks, which reduce the stress on the asphalt and ultimately enhance its low-temperature crack resistance.

### 3.3. Improvement of Rutting Resistance

**Figure 6:**
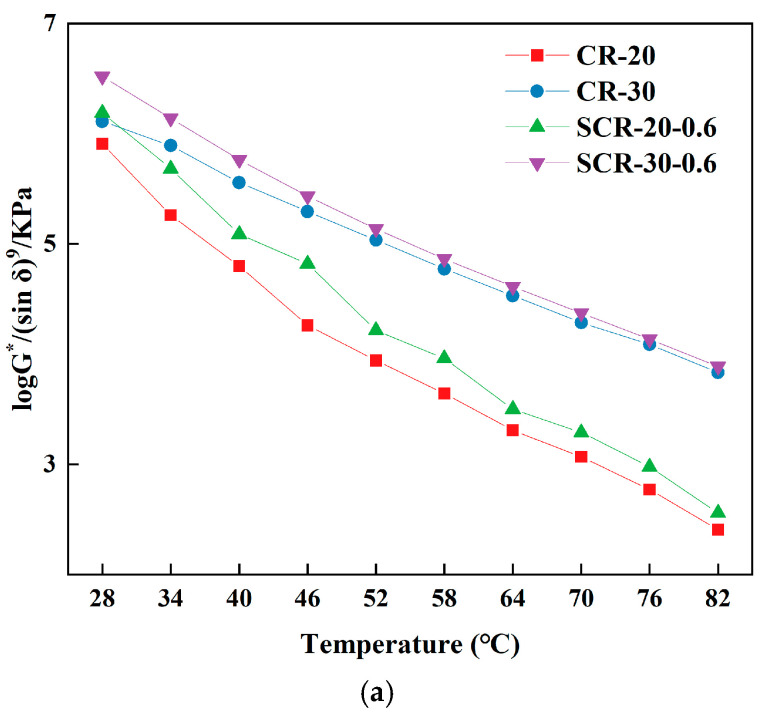

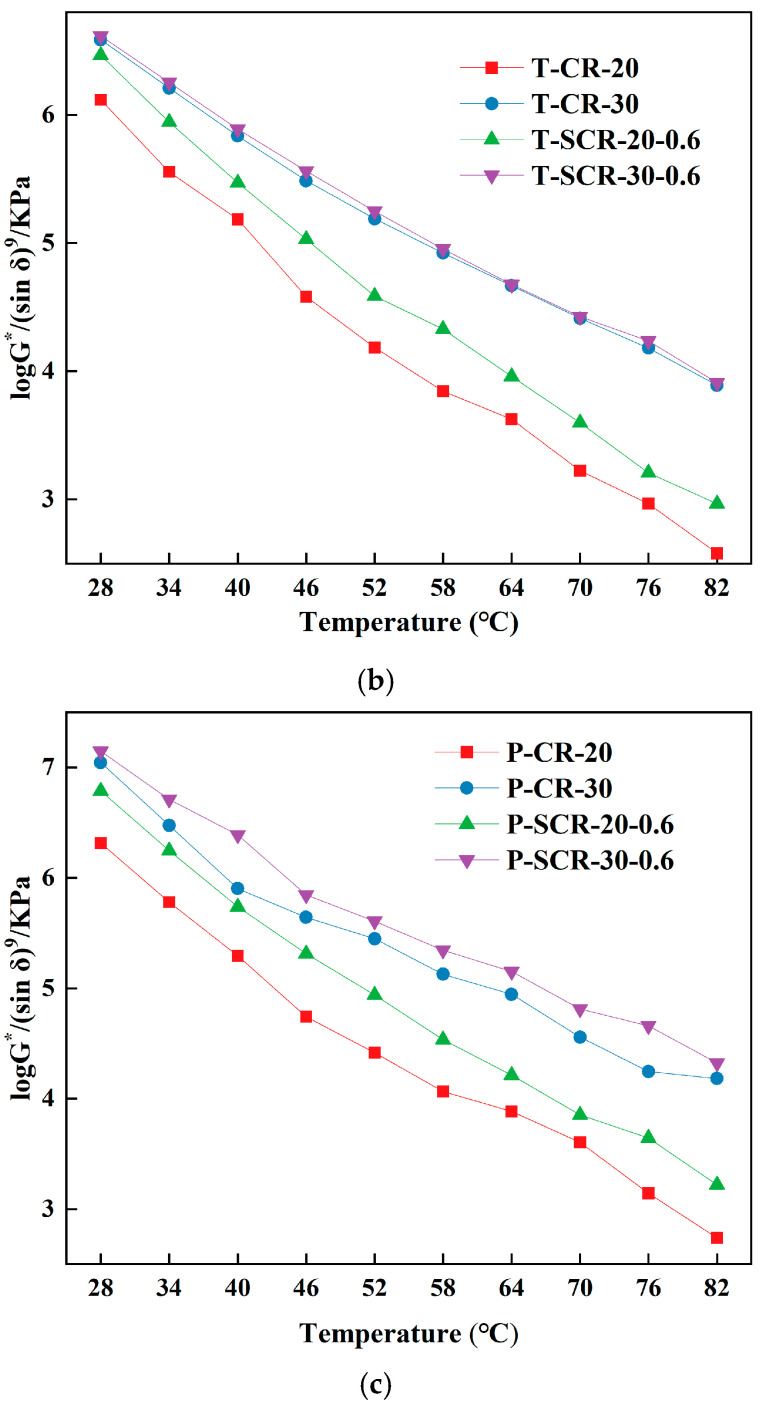
The improved rutting factor of high-rubber-content CRMA. (**a**) Pre-aging; (**b**) short-term aging; (**c**) long-term aging.

The high-temperature performance of asphalt specimens was evaluated using the G*/sin δ parameter specified in Section 3.1. The improved rutting factors of CR-20, CR-30, SCR-20-0.6, and SCR-30-0.6 are illustrated in Figure 6.

From Figure 6a, we can see that, under the test temperature, the improved rutting factors of CR-30 were greater than CR-20, and the improved rut factor of SCR-30-0.6 was also greater than that of SCR-20-0.6, indicating that the addition of rubber increased the improved rut factor. This is because rubber is a kind of polymer, with good elastic and viscoelastic characteristics; these characteristics increase in asphalt when more crumb rubber is incorporated into the mixture. The addition of rubber can increase the bonding ability and elastic modulus of the asphalt, so that the asphalt can better resist deformation at high temperatures. There are many pores in the asphalt, and these pores cause asphalt deformation at high temperatures. Crumb rubber has a small particle size (60 mesh) and high filling performance, which allows it to fill the pores in the asphalt, reducing its porosity and improving its high-temperature deformation resistance.

From Figure 6b,c, the improved rutting factors of asphalt specimens exhibit a gradual decline as the temperature increases under aging conditions. Among them, the improved rutting factor of SCR-30-0.6 after aging was the largest, indicating that the high-rubber-content CRMA with 0.6% SDYK warm-mixing agent had the strongest high-temperature deformation resistance with aging. Under the test temperature, the improved rutting factor of high-rubber-content CRMA was greater than ordinary-rubber-content CRMA after aging, indicating that the addition of more crumb rubber could improve the high-temperature deformation resistance of asphalt under aging conditions. After aging, the improved rutting factor of the CRMA with the SDYK was greater than that without the SDYK, indicating that the SDYK could improve the high-temperature deformation resistance of CRMA under aging conditions.

### 3.4. Quantitative Characterization of Chemical Functional Groups

**Figure 7:**
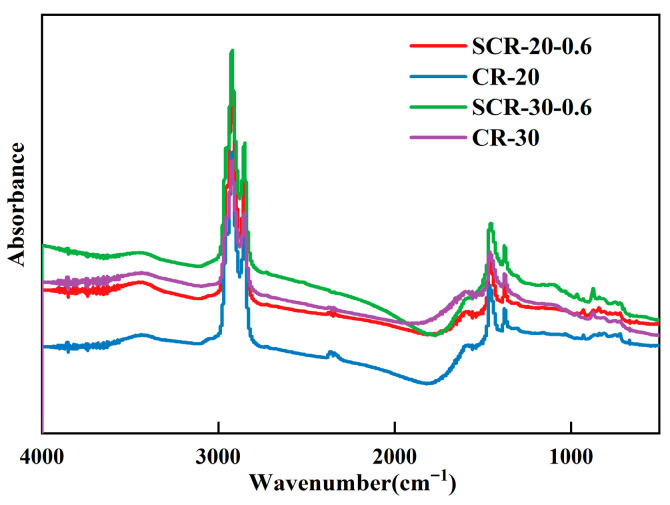
Infrared spectrum of CRMA.

The infrared spectra of CR-20, CR-30, SCR-20-0.6, and SCR-30-0.6 are shown in Figure 7. The absorption peak positions of asphalt are roughly the same. This observation indicates that the four asphalt types share fundamental similarities in their molecular composition and chemical functional groups. At the range of 2700–2950 cm^−1^, all four types of asphalt exhibit strong absorption peaks. This is because the C-H bond in saturated alkanes can cause asymmetric stretching vibration and symmetric stretching vibration, resulting in specific absorption peaks [51]. The absorption frequency of SCR-30-0.6 is higher than that of CR-30, and the absorption frequency of SCR-20-0.6 is higher than that of CR-20, indicating that SDYK reduces the segregation phenomenon. Strong absorption peaks also appear around 1310 cm^−1^ to 1490 cm^−1^, mainly due to the presence of alkanes, cycloalkanes, and a small amount of -NH inducing symmetric and asymmetric bending vibrations [46]. The four types of asphalt do not show characteristic peaks at wave numbers around 1650 cm^−1^ to 1750 cm^−1^. The characteristic peaks here are mainly due to the stretching vibration of carbon oxygen double bonds, while the rate of carbonyl generation is slow and the amount generated is relatively small. A low intensity absorption peak appeared at around 1600 cm^−1^, which was caused by the stretching vibration of the carbon double bond (C=C) [23,52].

**Figure 8:**
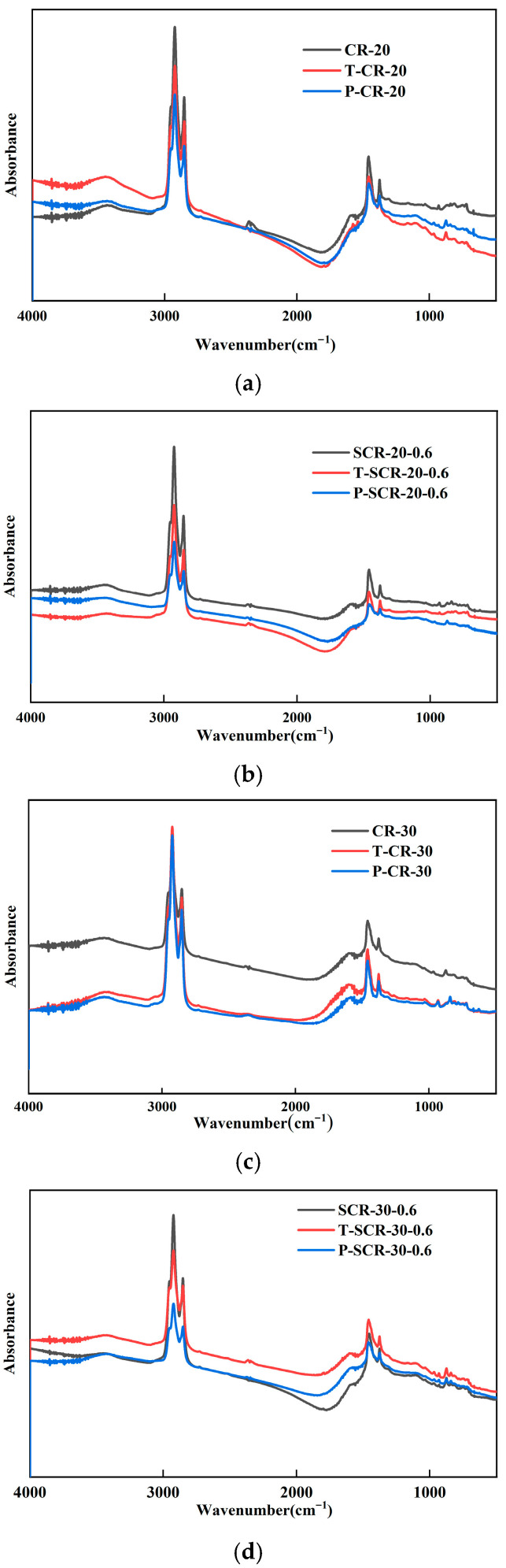
Infrared spectrum of aged CRMA. (**a**) CR-20; (**b**) SCR-20-0.6; (**c**) CR-30; (**d**) SCR-30-0.6.

The infrared spectra of aged CRMA are shown in Figure 8. The infrared spectra of asphalt remain roughly unchanged after aging. However, after aging, the intensity (area) of the characteristic peak of the sulfoxide group at around 970 cm^−1^ for the four types of asphalt significantly increased [53]. This is mainly caused by the stretching vibration of the sulfoxide group, S=O, which is due to the reaction between sulfur-containing compounds and oxygen in the asphalt material during aging. The carbonyl absorption peak at 1700 cm^−1^ significantly increased, mainly due to the stretching vibration of the carbonyl group. The absorption peak of asphalt also showed significant changes at around 1465 cm^−1^, indicating that the aging process affects the aromatic structure of asphalt. The higher the aromatic ring index, the more stable the structure of asphalt, and the better its high-temperature performance; this verifies the enhanced high-temperature deformation resistance of asphalt under aging, as discussed in Section 3.3. The absorption peak of asphalt increased at 1600 cm^−1^, which was caused by the vibration of the aromatic ring skeleton due to the substitution of polar groups on the aromatic ring in aromatic compounds [54]. The aromatic ring increases the rigidity of the molecular chain segment, leading to a decrease in fluidity. This finding indicates that the low-temperature cracking resistance of asphalt deteriorates with aging, which verifies the experimental results of BBR and DSR in this paper.

Infrared spectroscopy exhibits overlapping absorption peaks, leading to inherent limitations in the precise characterization of chemical functional group variations in asphalt. To address these issues, this study employed the Lambert–Beer law to calculate absorption peak parameters. These parameters were used for further analysis of the effects of warm-mix additives and aging on the chemical functional groups of asphalt. The Lambert–Beer law [55] is expressed as follows:

(1) $$A=lg\frac{1}{T}=abc$$

In the formula, *A* represents the absorbance spectrum; *T* represents the transmittance spectrum; *a* denotes the absorbance per unit concentration and thickness; *b* represents thickness; *c* represents concentration.

The functional group calculation formula for infrared spectroscopy was as follows [56]:

(2) $$I_{C=O}=\frac{A_{C=O}}{\sum A_{{2000\sim 600cm}^{-1}}}$$

(3) $$I_{S=O}=\frac{A_{S=O}}{\sum A_{{2000\sim 600cm}^{-1}}}$$

(4) $$I_{Aromaticity}=\frac{A_{{1598.36cm}^{-1}}}{\sum A_{{2000\sim 600cm}^{-1}}}$$

(5) $$I_{Aliphatic}=\frac{A_{{1459.98cm}^{-1}}+A_{{1375.63cm}^{-1}}}{\sum A_{{2000\sim 600cm}^{-1}}}$$

(6) $$I_{Branchedalkane}=\frac{A_{{1375.63cm}^{-1}}}{A_{{1459.98cm}^{-1}}+A_{{1375.63cm}^{-1}}}$$

In the formula, *A_C=O_* represents the area of the carbonyl characteristic peak at 1700 cm^−1^; *A_S=O_* represents the area of the sulfoxide group characteristic peak at 1032 cm^−1^; *A*_1600cm_^−1^ represents the aromatic ring peak area at 1600 cm^−1^; *A*_1460cm_^−1^ represents the aromatic ring peak area at 1460 cm^−1^; *A*_1377cm_^−1^ represents the aromatic ring peak area at 1377 cm^−1^; $\sum A_{{2000\sim 600cm}^{-1}}$ represents the total area of C–H characteristic peaks within the 2000–600 cm^−1^.

In this study, OMNIC 9.2 software and Origin2025 software were employed to calculate the peak areas in the FTIR spectra of asphalt. Subsequently, by applying Formulas (1)–(6), the chemical functional group indices of various asphalt samples were derived.

The chemical functional group indices of CRMA are shown in Table 4. The various chemical functional group indices of CR-20 are all less than those of SCR-20-0.6, and the various chemical functional group indices of CR-30 are all less than those of SCR-30-0.6. This indicates that the addition of SDYK increases the aromatic ring index, the aliphatic chain index, and the branched chain alkane index of asphalt. It also shows an increase in the content of saturated phenols and aromatic compounds in the asphalt, and a more even dispersion of asphaltenes within it, resulting in a more stable molecular structure of the asphalt. The branched chain alkane index of CR-30 is lower than that of CR-20, and the branched chain alkane index of SCR-30-0.6 is lower than that of SCR-20-0.6. This indicates that increasing the dosage of rubber reduces the fluidity of asphalt and increases its viscosity [57]. This is due to the interaction between the polymer compounds in the crumb rubber and the macromolecules in the asphalt, forming a tight three-dimensional mesh structure through chemical bonding or physical adsorption [58]. The formation of this three-dimensional mesh structure increases the interaction force between asphalt molecules, leading to a decrease in the fluidity of asphalt and an increase in its viscosity [58].

The addition of SDYK reduces the change rate in the carbonyl index, the sulfoxide index, and the aromatic ring index of asphalt under aging, so it shows that the addition of SDYK warm-mix agent improves the aging resistance of asphalt. The reason for this is that the molecules of the SDYK warm-mix agent break and distribute in the asphalt, hindering the oxidation reaction of oxygen and sulfur [59]. After short-term aging and long-term aging, the increase or decrease rates of the chemical functional groups in CR-30 are lower than those in CR-20, and the increase or decrease rates of the chemical functional groups in SCR-30-0.6 are lower than those in SCR-20-0.6. This is because high-rubber-content CRMA contains more network structures than ordinary content CRMA, which increases the intermolecular forces between asphalt molecules and makes the asphalt structure more stable.

### 3.5. Nanoscale Morphology and Mechanical Characterization

**Figure 9:**
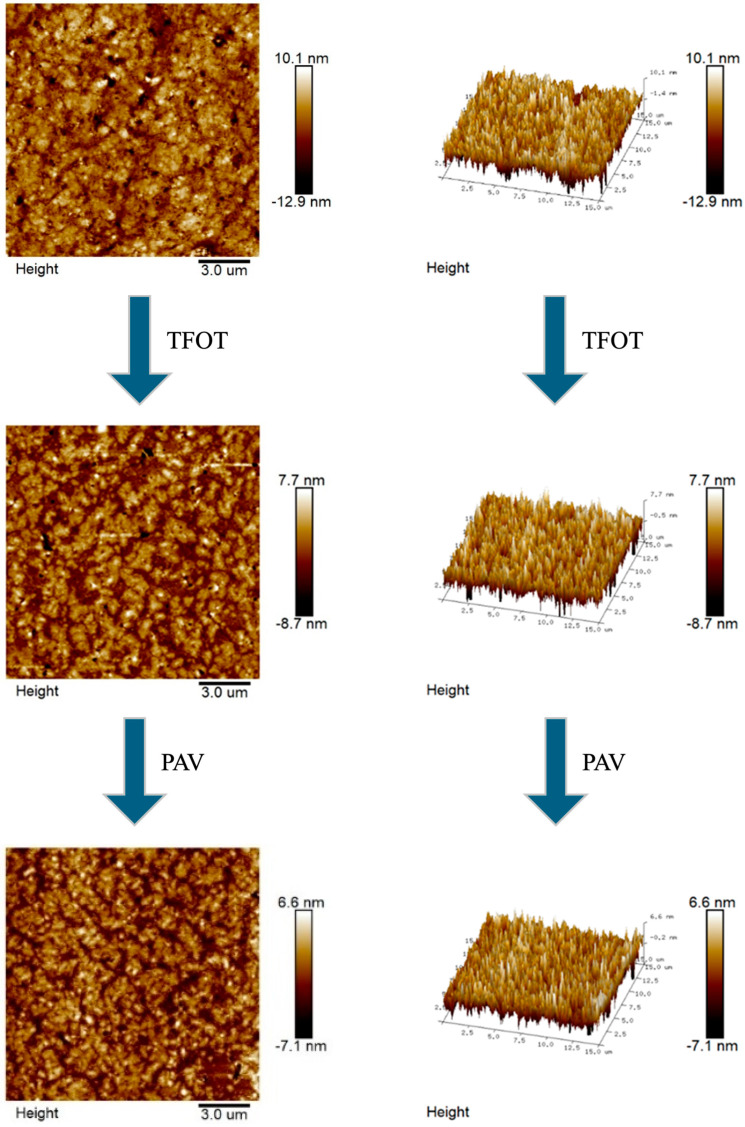
Microscopic and 3D representations of the CR-20 aging process.

**Figure 10:**
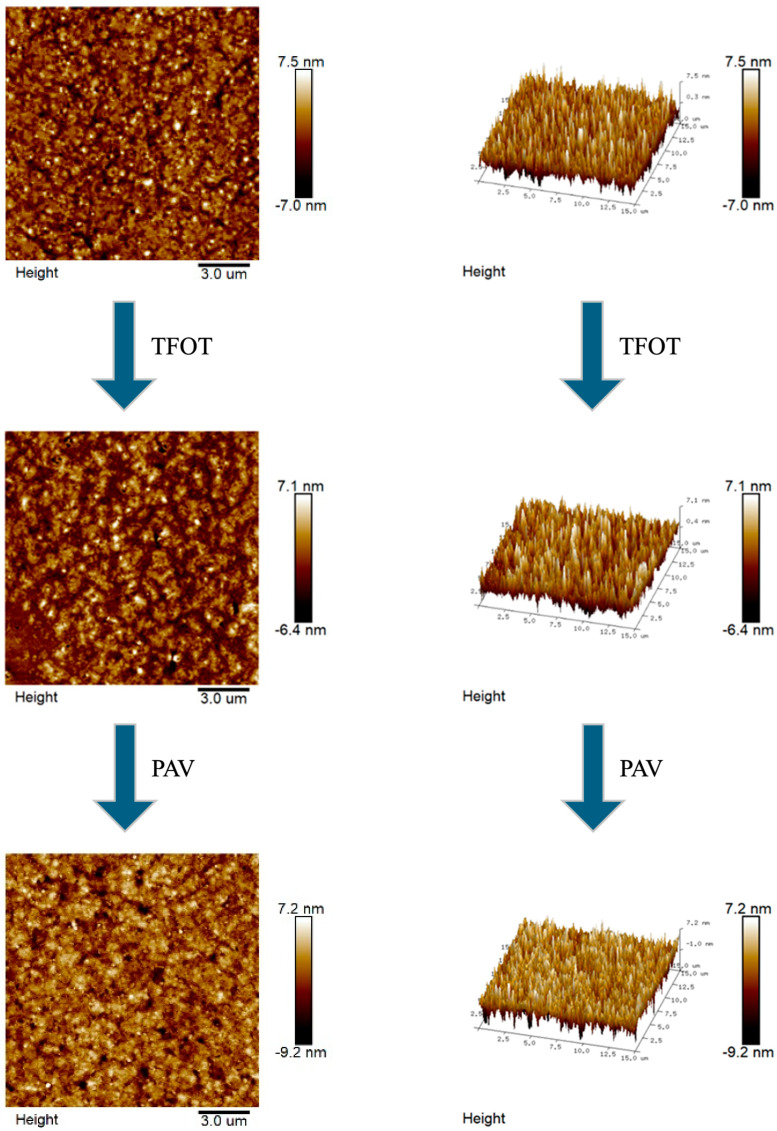
Microscopic and 3D representations of the SCR-20-0.6 aging process.

**Figure 11:**
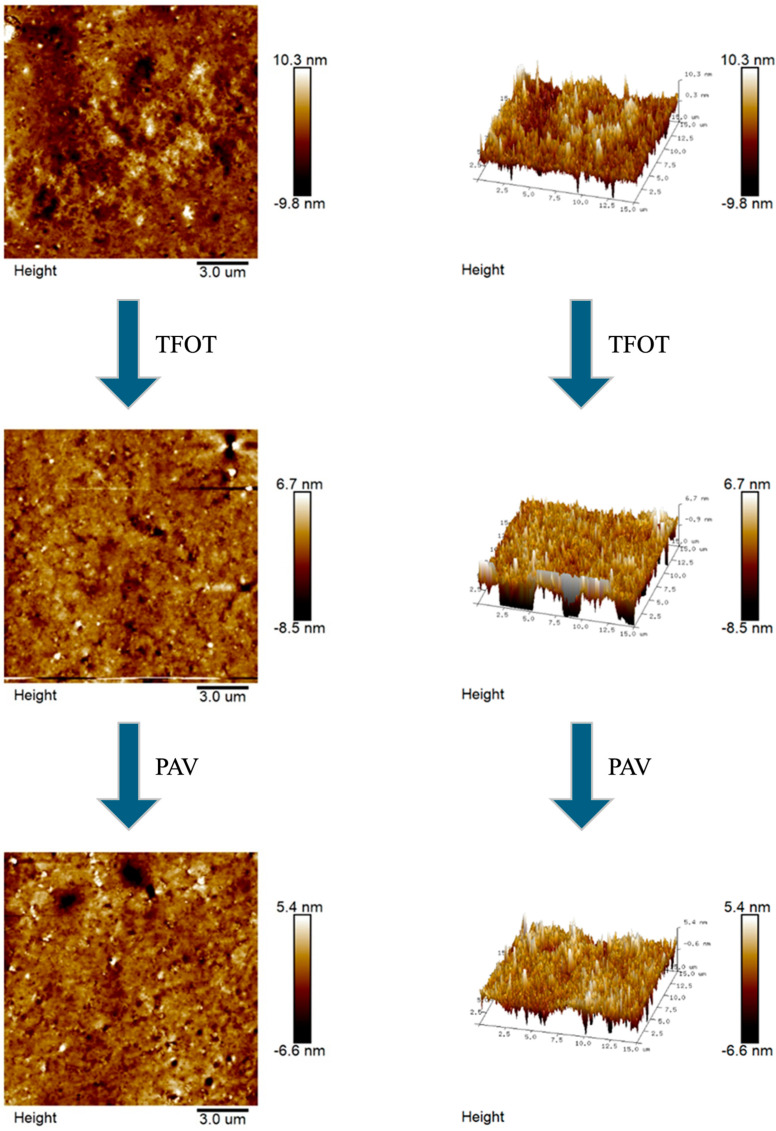
Microscopic and 3D representations of the CR-30 aging process.

**Figure 12:**
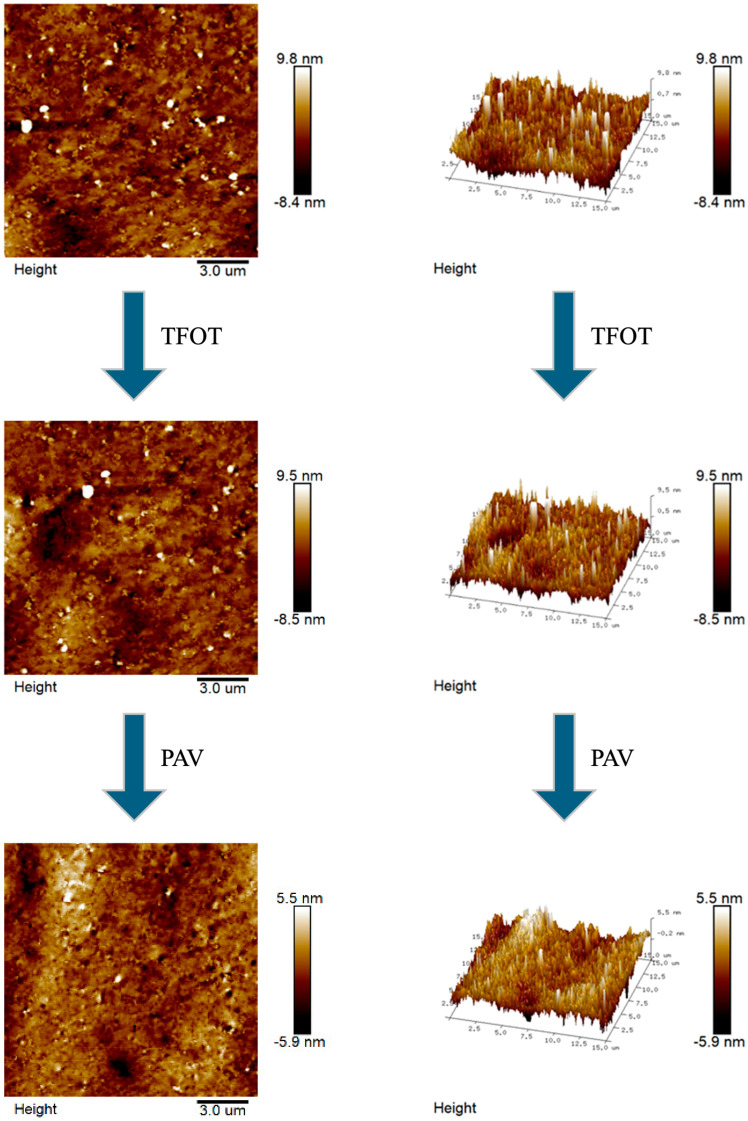
Microscopic and 3D representations of the SCR-30-0.6 aging process.

AFM enables the acquisition of microstructural topography and quantitative mechanical characterization of asphalt at the nanoscale. The microscopic morphology and 3D images of the aging process of CRMA were presented in Figure 9, Figure 10, Figure 11 and Figure 12.

In Figure 9, Figure 10, Figure 11 and Figure 12, we can see that there is no obvious “honeycomb” structure in the original, short-term aged, and long-term aged asphalts, but a unique “black and white alternating structure” appeared. This was because the light components in the asphalt are absorbed by the crumb rubber, and a gel film with high asphaltene content formed on the asphalt’s surface [60]. Due to the close connection between crumb rubber particles, it is difficult for them to form a “honeycomb-like” structure. The highest peaks of CR-20, SCR-20-0.6, CR-30, and SCR-30-0.6 were 10.1 nm, 75 nm, 10.3 nm, and 9.8 nm, respectively. The highest peak of CR-20 was higher than that of SCR-20, and the highest peak of CR-30 was higher than that of SCR-30-0.6. And from the 3D graph, it can be observed that the peak area of SCR-20-0.6 was more evenly distributed compared to that of CR-20, and the peak area of SCR-30-0.6 was more evenly distributed than that of CR-30. This was because the hydrophilic and oleophilic groups contained in SDYK had a lubricating effect on the surface of the asphalt, effectively preventing the aggregation of asphalt particles [61], and maintaining good dispersibility and flowability characteristics in the asphalt. As the aging degree deepens, the number of black and white alternating structures does not change much. The reason was that the surface structure of the crumb rubber changed from rough to smooth during the aging process, and its molecular chain segments allowed some smaller polymer molecules to enter the asphalt, promoting the absorption of light components and wax components [62]. In addition, the swelling rate of the crumb rubber accelerated, the distance between the crumb rubber molecules increased, reducing the cross-linking of the crumb rubber molecules, and the large and long straight chain molecules were more evenly distributed in the asphalt.

The surface roughness of the asphalt is closely related to the nanoscale structure distribution, characterized by peaks and valleys. Typically, the variance of the measured data’s height deviations is used to evaluate the average surface roughness of a sample. However, due to the subtle undulations and complex peak-valley intercrossing in the microstructure of asphalt surfaces, the root mean square roughness (*R_q_*) is selected to characterize the surface roughness of warm-mix CRMA. The greater the surface roughness, the more significant the phase separation in asphalt. This also indicates poorer microstructure stability, reduced elastic properties, and decreased high-temperature deformation resistance [48]. The definition of root mean square roughness is shown in Equation (7):

(7) $$R_{q}=\sqrt{\frac{\iint {\left[h\left(x,y\right)-h_{0}\right]}^{2}dA}{\iint dA}}$$

In the formula, A represents the scanning area; in this study, the scanning area is 20 × 20 μm^2^; h (x, y) denotes the topographical height function at the grid point (x, y); *h*_0_ represents the reference height, defined as $h_{0}=\frac{\iint h(x,y)dA}{\iint dA}$, where the integration is performed over the scanning area; *Rq* is expressed in nanometers (nm).

**Figure 13:**
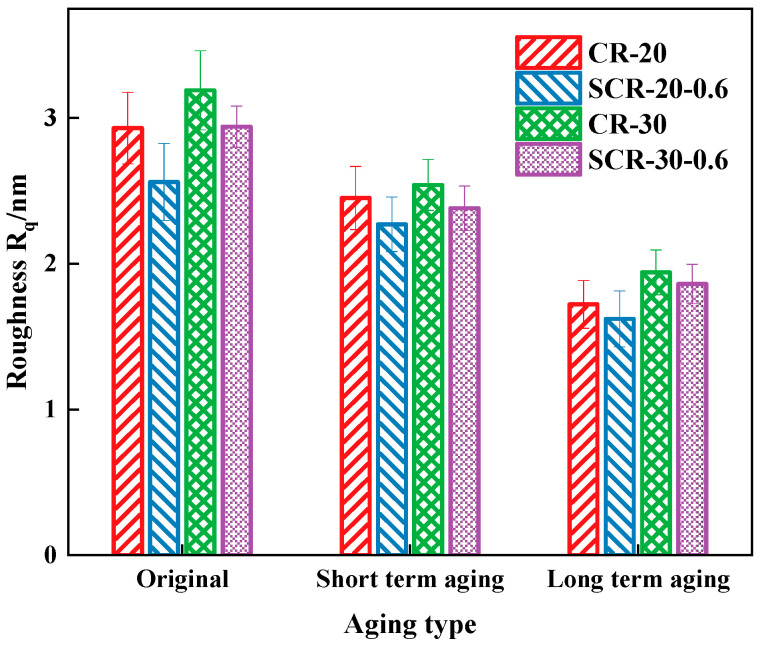
The surface roughness of four types of aged CRMA.

During the AFM testing process, the scanning area was 20 μm × 20 μm. Each sample was divided into four small regions, with four points scanned in each region. The data obtained were processed and analyzed using the “Roughness” module of the NanoScope Analysis 3.0 software to calculate the root mean square roughness (Rq) of both CRMA and warm-mix CRMA. The calculation results are shown in Figure 13.

From Figure 13, the roughness of asphalt gradually decreases after aging, and the phase differences at the micro scale are small, with obvious phase aggregation. Therefore, the structural stability of asphalt is strong, and macroscopically, it is manifested as enhanced high-temperature deformation resistance. This is because, as the aging degree deepens, the proportion of asphalt and other substances increases. The roughness of SCR-30-0.6 is lower than that of CR-30, and the roughness of SCR-20-0.6 is lower than that of CR-20, indicating that the addition of SDYK reduces the roughness of the asphalt. This is because SDYK is composed of polar and non-polar components, and the water film formed during the asphalt preparation process lubricates the asphalt structure, making the grid structure distribution of CRMA more uniform. The roughness of CR-30 is higher than that of CR-20, and the roughness of SCR-30-0.6 is higher than that of SCR-20-0.6, indicating that increasing the content of rubber will increase the roughness of the asphalt. This occurs because a higher amount of crumb rubber, with its higher molecular weight, becomes more evenly distributed in the asphalt, promoting more cross-linking and focal points, and thus creating a more complex network and rougher surface. It is observed that the roughness of CR-30 surpasses that of SCR-20-0.6, indicating that the addition of greater quantities of crumb rubber has a more pronounced effect on the asphalt’s roughness compared to adding SDYK.

The QNM (Quantitative Nanomechanical Mapping) mode of AFM was employed to analyze the mechanical properties of high-rubber-content CRMA under aging conditions. The analysis included adhesion force, DMT modulus, and energy dissipation. The formulas for calculating adhesion force and DMT modulus are as follows [63,64]:

(8) $$F_{NM}=\theta \times {∆}_{x}\times K$$

(9) $$F_{tip}-F_{NM}=\frac{4}{3}E^{*}\sqrt{R}\delta^{\frac{3}{2}}$$

(10) $$E^{*}={\left(\frac{1-V_{S}^{2}}{E_{S}}+\frac{1-V_{tip}^{2}}{E_{tip}}\right)}^{-1}$$

In the equation, *F_NM_* indicates the adhesion force between the probe and the asphalt surface; θ is the correction factor; ${∆}_{x}$ refers to the deflection of the probe cantilever; *K* is the elastic constant of the probe cantilever; *F_tip_* represents the applied force of the probe on the asphalt surface; E^*^ is the reduced DMT modulus of asphalt; *R* denotes the tip radius of curvature; δ represents the deformation of asphalt; *V_S_*, *V_tip_* are the Poisson’s ratios of asphalt and the probe, respectively; *E_S_*, *E_tip_* are the Young’s moduli of asphalt and the probe, respectively.

**Figure 14:**
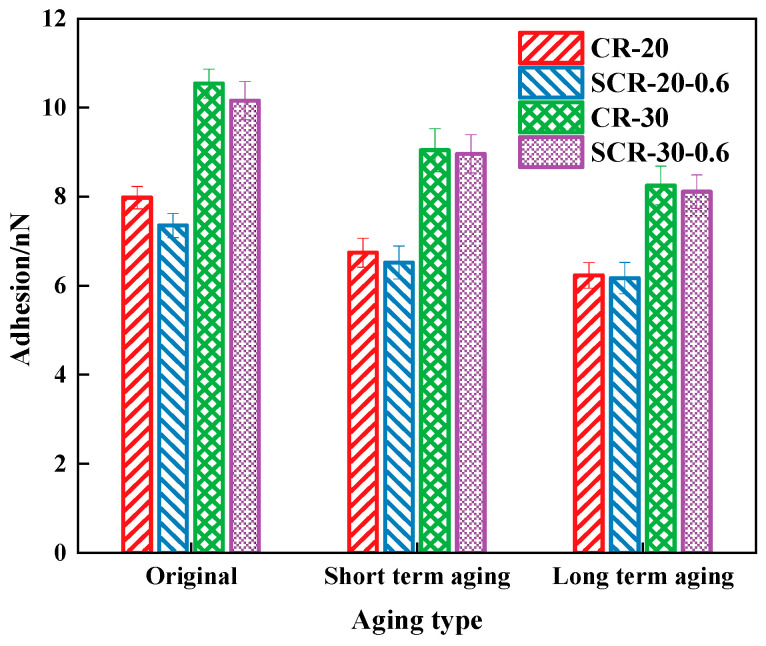
Adhesion of four types of aged CRMA.

The adhesion of four types of CRMA before and after aging is presented in Figure 14. From Figure 14, we can see that the adhesion force of SCR-20-0.6 is lower than that of CR-20, and the adhesion force of SCR-30-0.6 is lower than that of CR-30. This is because the water film formed by SDYK during the asphalt preparation process lubricates the asphalt structure, resulting in a more uniform distribution of the grid structure of the CRMA, a reduced phase separation phenomenon, decreased roughness, and a reduced adhesion area. Therefore, adding SDYK results in a reduction in the adhesion between asphalt and aggregates. Among the types tested, CR-30 exhibits a higher adhesion force compared to CR-20, and SCR-30-0.6 shows greater adhesion than SCR-20-0.6. Therefore, increasing the amount of crumb rubber enhances asphalt adhesion. This occurs due to the increased surface roughness created by the crumb rubber, which improves the bonding between the asphalt and the aggregate. As a result, the high adhesion contributes to better low-temperature crack resistance in asphalt mixtures. Moreover, this affirms that raising the crumb rubber content, as discussed earlier, can also improve the low-temperature cracking resistance. The lightweight components of all four types of asphalt decrease, and as the aging degree deepens, the adhesion of asphalt decreases.

This is because aging increases the weight of heavy molecules in asphalt, and the viscous components in asphalt decrease accordingly [65]. After adding SDYK, the adhesion of the asphalt decreased after aging.

**Figure 15:**
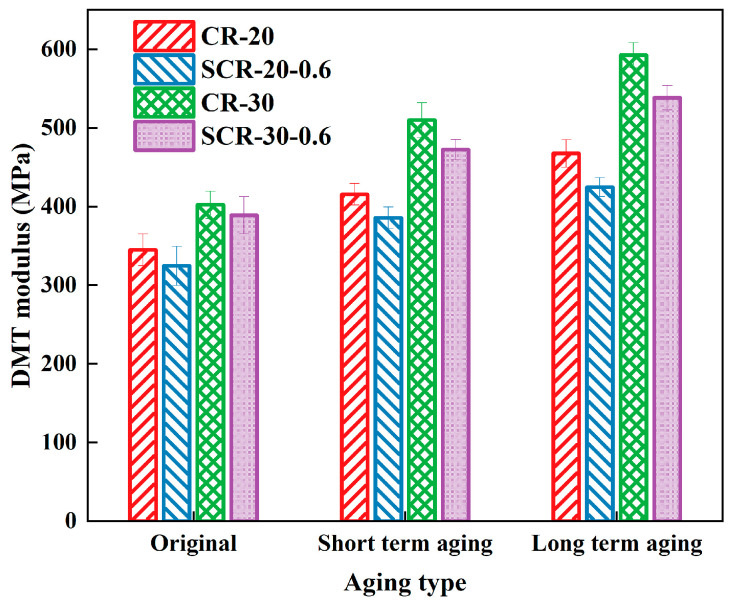
DMT modulus of four types of aged CRMA.

The DMT modulus of CRMA before and after aging is illustrated in Figure 15. From Figure 15, the DMT modulus of CR-20 is higher than that of SCR-20-0.6, and the DMT modulus of CR-30 is higher than that of SCR-30-0.6, indicating that the addition of SDYK reduces the DMT modulus of asphalt. This is because the addition of SDYK has a lubricating effect on the asphalt structure, making its surface roughness smaller and more flowable; that is, it has better deformation ability at low temperatures.

This also confirms that the addition of SDYK mentioned earlier can improve the high-temperature deformation resistance of asphalt. The DMT modulus of CR-30 exceeds that of CR-20, and SCR-30-0.6 shows a higher DMT modulus than SCR-20-0.6, indicating that adding crumb rubber increases the DMT modulus of asphalt, thereby improving its deformation resistance. Furthermore, as the degree of aging increases, the DMT modulus of all four asphalt types rises. This is attributed to aging, which increases the heavy molecular weight of the asphalt, affecting the DMT modulus and significantly improving its high-temperature deformation resistance.

## 4. Conclusions

This study demonstrates that high-rubber-content CRMA has significantly enhanced performance based on multi-scale evaluation of its rheological deterioration and micromechanical characteristics. A comparative analysis was conducted between high-rubber-content and conventional-rubber-content CRMA under aging conditions. The main conclusions of this study are as follows:

(1) Comparing the high and low temperature performance of warm-mixed high-rubber-content CRMA with different SDYK contents, the findings show that the optimal SDYK warm-mix agent dosage for high-rubber-content CRMA is 0.6%.

(2) SCR-30-0.6 exhibited superior high- and low-temperature performance and anti-aging performance. The addition of increased amounts of crumb rubber and the warm-mix agent improved the cracking and rutting resistance performance.

(3) Compared with CR-20, SCR-30-0.6 showed an increase of approximately 13.42% in the aliphatic index and 19.81% in the branched alkane index. This indicates that the warm-mix agent and rubber additive enhanced the aging resistance of the asphalt.

(4) The distinct phase-separated morphology led to a “black-and-white alternating structure” rather than the conventional “honeycomb” patterns, due to addition of rubber. The addition of SDYK warm-mix agent led to a decrease in the roughness, DMT modulus, and adhesion of asphalt. Increasing the dosage of crumb rubber will increase the roughness, adhesion, and DMT modulus of asphalt.

(5) The SDYK warm-mix additive produced a lubricating effect, reducing the viscosity of the asphalt, and thereby enabling higher rubber content while maintaining good workability at lower temperatures.

In summary, the integration of warm-mix additives and high rubber content effectively improved the performance of traditional rubberized asphalt, including its aging resistance and high- and low-temperature performances. This work provides a foundation for the practical use of sustainable, high-performance CRMA in regions with large temperature fluctuations.

## Figures and Tables

**Table 1:** Physical performance indices of 60 mesh rubber particles.

Technical Index	Technical Requirement	Test Result
Volume density g/cm^3^	1.15 ± 0.05	0.89
Ash content%	≤8	6
Iron content%	≤0.03	0.021
Heating decrement%	≤1	0.6
Fiber content%	<1	0.4

**Table 2:** Types and representative symbols of test materials.

Asphalt Types	Symbols
Asphalt with 20% crumb rubber content	CR-20
Asphalt made by adding SDYK with 20% crumb rubber content	SCR-20-0.6
Asphalt with 30% crumb rubber content	CR-30
Asphalt made by adding 0.4% SDYK with 30% crumb rubber content	SCR-30-0.4
Asphalt made by adding 0.6% SDYK with 30% crumb rubber content	SCR-30-0.6
Asphalt made by adding 0.8% SDYK with 30% crumb rubber content	SCR-30-0.8
Short-term aging (TFOT)	T
Long-term aging (PAV)	P

**Table 3:** Technical indices of asphalt.

Technical Index	Penetration at 25 °C, 100 g, 5 s, 0.1 mm (mm)	Softening Point (°C)	Ductility at 5 °C (mm)	Viscosity at 175 °C (Pa·s)
Specification	ASTM D5	ASTM D36	ASTM D113	ASTM D4402
Pen 90 base asphalt	91.5	51.5	94	0.3
CR-30	50.8	78.3	209	3.1
SCR-30-0.4	49.5	90.9	217	3.0
SCR-30-0.6	49.1	92.7	221	2.8
SCR-30-0.8	49.3	91.2	219	2.9

**Table 4:** Chemical functional group indices of aged CRMA.

Asphalt Types	Carbonyl Index	Sulfoxide Index	Aromatic Ring Index	Aliphatic Index	Branched Chain Alkane Index
CR-20	0.00193	0.00305	0.00072	0.723	0.106
T-CR-20	0.00216	0.00324	0.00089	0.637	0.090
P-CR-20	0.00233	0.00347	0.00097	0.623	0.086
SCR-20-0.6	0.00228	0.00365	0.00166	0.895	0.147
T-SCR-20-0.6	0.00250	0.00387	0.00199	0.814	0.141
P-SCR-20-0.6	0.00264	0.00404	0.00222	0.793	0.139
CR-30	0.00217	0.00320	0.00079	0.655	0.104
T-CR-30	0.00239	0.00334	0.00096	0.586	0.089
P-CR-30	0.00251	0.00355	0.00103	0.567	0.085
SCR-30-0.6	0.00322	0.00395	0.00167	0.820	0.127
T-SCR-30-0.6	0.00325	0.00411	0.00195	0.788	0.122
P-SCR-30-0.6	0.00342	0.00433	0.00209	0.779	0.121

## Data Availability

The original contributions presented in this study are included in the article. Further inquiries can be directed to the corresponding authors.
